# What are the most important research questions within prediabetes? A priority setting partnership in collaboration with patients, healthcare professionals and researchers

**DOI:** 10.1007/s00125-025-06505-4

**Published:** 2025-08-04

**Authors:** Amalie K. Andersen, Kristian D. Lyng, Kristine Færch, Dorte Vistisen, Bernt J. von Scholten, Michael S. Rathleff, Janus L. Thomsen, Morten H. Jensen

**Affiliations:** 1 Department of Health Science and Technology, Aalborg University, Aalborg, Denmark; 2 Center for General Practice at Aalborg University, Aalborg University, Aalborg, Denmark; 3 Data Science, Novo Nordisk A/S, Søborg, Denmark; 4 Steno Diabetes Center Copenhagen, University of Copenhagen, Herlev, Denmark

**Keywords:** Patient and public involvement, Prediabetes, Research priorities

## Abstract

**Aims/hypothesis:**

Research agendas are typically set by researchers and funders, meaning that priorities of end users, such as patients and healthcare professionals (HCPs), could be missed or overlooked in research. To ensure future research in prediabetes is of relevance and benefit to people with prediabetes and HCPs, it is important to involve these stakeholders in setting the research agenda. The aim of this study was to establish a top-10 list of the most important research questions in prediabetes (HbA_1c_ 42–47 mmol/mol [6.0–6.4%]) by involving and collaborating with patients, relatives, patient organisations, HCPs and researchers.

**Methods:**

We used a modified James Lind Alliance Priority Setting Partnership methodology, following the four-step process including: (1) Gathering uncertainties; (2) Organising uncertainties; (3) Interim priority setting; and (4) Final priority setting in a workshop. Further, the international relevance of the final top-10 list was assessed.

**Results:**

A total of 1142 responses were submitted by 405 people to: ‘What questions about prediabetes would you like to see answered by research?’. The collected uncertainties were categorised and condensed into 35 indicative questions. Through prioritisation, patients and relatives had different preferences from researchers and HCPs. The jointly agreed top-10 list included questions on prevention strategies, risk factors, diet advice, screening and personalised treatment. Highest prioritisation was given to: ‘What is the best prevention of diabetes and will early prevention strategies reduce the number of people with type 2 diabetes?’.

**Conclusions/interpretation:**

An iterative and collaborative process identified shared priorities between patients, HCPs and relevant stakeholders in prediabetes. Findings should support academia, funders and the healthcare industry to target research within prediabetes specifically to the needs of patients and HCPs.

**Graphical Abstract:**

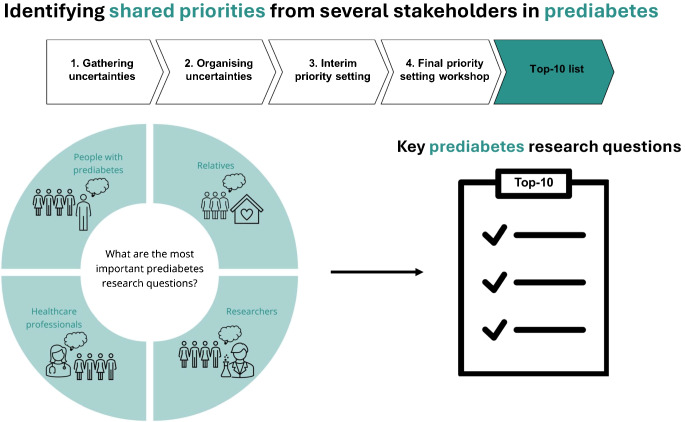

**Supplementary Information:**

The online version contains peer-reviewed but unedited supplementary material available at 10.1007/s00125-025-06505-4.


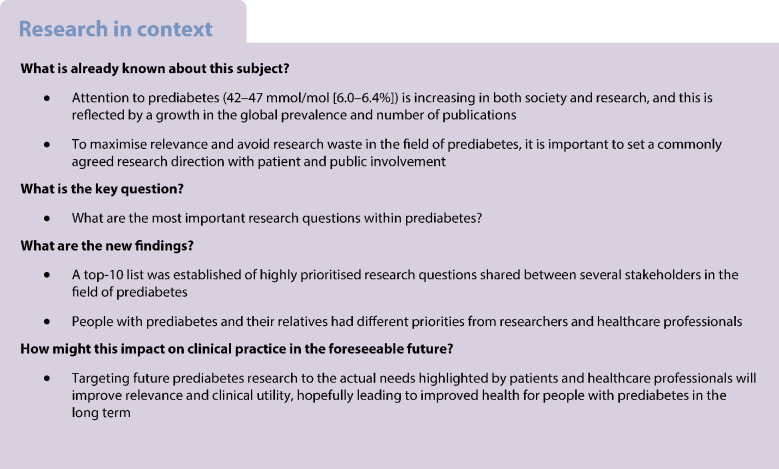


## Introduction

During the past decade, attention to prediabetes has increased in both society and research [1, 2]. Prediabetes is a condition where the blood glucose levels are higher than normal, but not high enough to be above the threshold for type 2 diabetes (HbA_1c_ >48 mmol/mol [>6.5%]) [3–5]. There is no global consensus for the diagnostic criteria for prediabetes since different thresholds and tests are currently used. These tests include HbA_1c_, fasting plasma glucose and 2 h plasma glucose. Based on HbA_1c_, the International Expert Committee (IEC) and National Institute for Health and Care Excellence (NICE) recommend prediabetes to be in the range 42–47 mmol/mol (6.0–6.4%), whereas the ADA recommendation is 39–47 mmol/mol (5.7–6.4%) [3, 6, 7]. The prevalence of HbA_1c_-defined prediabetes was estimated to be 12.3% (ADA threshold) and 4.3% (IEC threshold) in the US population in 2016 [8]. In addition, the global prevalence of prediabetes based on impaired fasting glucose and impaired glucose tolerance was estimated to be 5.8–9.1% in 2021 and estimates projected for 2045 indicate an increase to 6.5–10.0% [2]. Typically, no physical symptoms are present, meaning that most people are not aware of having prediabetes. A report by the Centers for Disease Control and Prevention (CDC) showed that only 19% of people with prediabetes in the US population were told by a healthcare professional (HCP) that they had prediabetes [9]. However, 25% of people with HbA_1c_-defined prediabetes will progress to type 2 diabetes within 5 years and some people with prediabetes are at high risk of developing long-term micro- or macrovascular complications [10–12]. Thus, prediabetes potentially generates a large burden on the individual as well as on society and the healthcare system.

To improve the management and health of people living with prediabetes, it is crucial to integrate perspectives from individuals with prediabetes, relatives, HCPs and the healthcare industry when setting the research agenda. Patient and public involvement in health research is believed to improve the quality, relevance and clinical utility of the research. Previously, patients, HCPs and relevant stakeholders have successfully been involved in identifying research priorities in other healthcare research fields, e.g. within type 1 diabetes, early cancer detection and type 2 diabetes [13–15]. However, involvement of patients and the public is still lacking in many areas, since scientific research agendas are often set by researchers and funders, which may ultimately compromise patient and public priorities in the research agendas [16–18].

A commonly agreed direction for prediabetes research is yet to be established with patient and public involvement. Additionally, prediabetes is a young research field with an upward trend in number of publications, which makes it even more important to set a research direction to maximise relevance and to avoid research waste [1, 19]. Therefore, the aim of this study was to identify a jointly agreed list of the most important research priorities within prediabetes established between people with prediabetes, relatives, HCPs and researchers.

## Methods

**Fig. 1:**
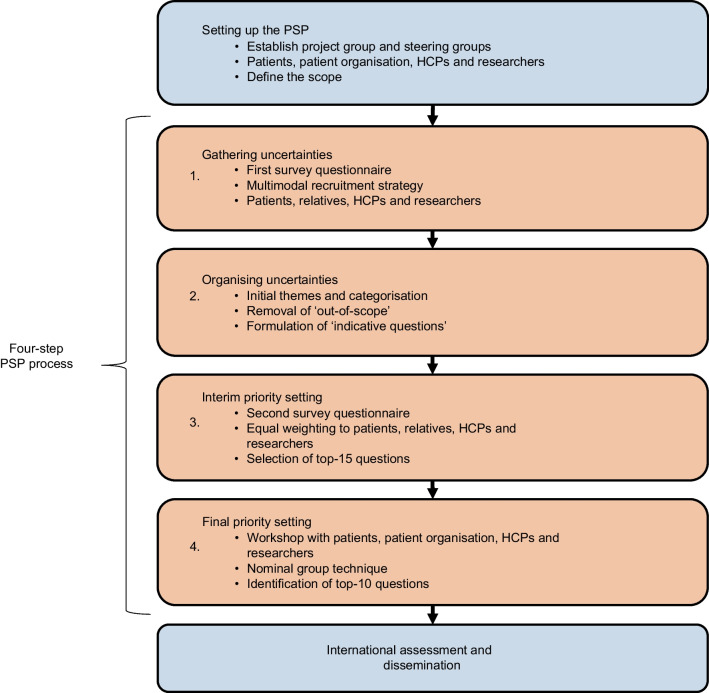
Flow chart showing the four-step PSP process. ‘Patients’ represents people living with prediabetes and people living with type 2 diabetes

The study was conducted in Denmark based on a modified version of the James Lind Alliance (JLA) Priority Setting Partnership (PSP) methodology [20]. JLA PSP is a standardised methodology used to bring patients, their relatives, HCPs and other key stakeholders together to identify the most important uncertainties within a research field [20]. The study followed the four-step PSP process: (1) Gathering uncertainties; (2) Organising uncertainties; (3) Interim priority setting; and (4) Final priority setting (Fig. 1). Additionally, an assessment was conducted to evaluate the top-10 priorities’ international relevance. The modification of the original PSP methodology is described in detail below. In each step, patients, relatives, HCPs and researchers were involved. People with prediabetes (HbA_1c_ 42–47 mmol/mol [6.0–6.4%] according to IEC recommendations) and people with type 2 diabetes (HbA_1c_ ≥48 mmol/mol [>6.5%]) were included as patients, and general practitioners, nurses and dietitians involved in the treatment and/or management of individuals with prediabetes or type 2 diabetes were included as HCPs. We included people with type 2 diabetes in the study because we believe they would be able to reflect on topics they would have liked to know before being diagnosed with type 2 diabetes. Furthermore, relatives (family members or friends) of people with prediabetes or type 2 diabetes and researchers working with prediabetes or type 2 diabetes were included.

The study has been approved by the local ethics committee at Aalborg University (case number: 2023-505-00137).

### Setting up the PSP

Initially, two steering groups and a project group were established. Steering group 1 consisted of ten people (eight researchers and two HCPs) and Steering group 2 consisted of three people (one person with prediabetes, one person with type 2 diabetes and a representative from the Danish Diabetes Association [patient organisation]). The steering groups provided input into the protocol and participated in each step of the PSP process. The project group consisted of eight people and was responsible for formulating the protocol, conducting the study and publishing the results. The study protocol was approved by both steering groups and pre-registered at Open Science Framework (10.17605/OSF.IO/GCQJY).

### Step 1: Gathering uncertainties from a national survey

An online survey questionnaire (electronic supplementary material [ESM] Fig. 1) was constructed to collect research uncertainties within prediabetes. The primary aim of the survey was to collect answers to: ‘What questions about prediabetes would you like to see answered by research?’. Each participant was able to submit between one and five questions in open-text fields. Second, the survey collected basic demographics (age, gender, geography, diabetes duration, type of HCP and educational level) of the participants. This information was used to identify possible under-represented groups, ensuring that perspectives were collected from a broad range of people. Lastly, all participants were asked whether they would like to participate in later steps of the PSP process. All survey data were collected through REDCap (Research Electronic Data Capture) hosted at Aalborg University [21].

The survey questionnaire was constructed in an iterative process by both steering groups and was pilot-tested by five people before distribution. The survey questionnaire was distributed using a multimodal recruitment strategy to all geographical areas of Denmark through social media (private groups and targeted advertisement through Facebook), personal networks (email and mouth-to-mouth) and relevant newsletters. On these online platforms a link or QR code directed participants to the survey, which relied on participants to self-report their status as: person with prediabetes, person with type 2 diabetes, relative, HCP or researcher. Furthermore, we recruited participants having a confirmed biochemical measure of prediabetes identified through previously conducted clinical trials [22, 23]. These participants had given consent to be contacted by mail with an invite to voluntarily participate in future studies. Sensitivity analyses were performed separately for participants with prediabetes and type 2 diabetes to assess if the responses were different between the two populations.

### Step 2: Organising uncertainties

The collected uncertainties from step 1 were analysed and organised into themes. NVivo (version 14.23.2; https://lumivero.com/products/nvivo/) was used for the qualitative data analysis, which was conducted by the project group. Initially, all uncertainties were systematically reviewed and interpreted based on naive reading and initial themes were identified for each uncertainty iteratively as the uncertainties were processed. Uncertainties gathered from responses where participants had submitted more than one uncertainty in a single entry were separated. Furthermore, out-of-scope and unidentifiable uncertainties were removed at this stage. For the out-of-scope assessment, an uncertainty was removed if the uncertainty was not about management of prediabetes. Additionally, unidentifiable uncertainties were categorised into three subgroups: missing information, incomplete row (e.g. repetition of letters or words that do not form a complete sentence) and personal healthcare issue (including questions that were too person-specific). The study deviated from the JLA PSP methodology by not evaluating uncertainties as ‘true uncertainties’, thus not removing uncertainties for being ‘already answered’ by research [20]. This was expected to result in a less biased organisation of the uncertainties. Two independent reviewers (one researcher and one HCP) were used to verify the removed uncertainties to minimise bias and improve reliability of the qualitative analysis. After organising the uncertainties into initial themes, these were further thematised into main and sub-themes aiming to lower the number of themes. Sub-themes mentioned by less than ten original uncertainties were removed. Following this, the uncertainties were used to formulate an indicative question for each sub-theme. For each indicative question, the original uncertainties and number of times the sub-theme was identified by each group was documented. Lastly, before going to prioritisation of the indicative questions, all questions were discussed and verified by both steering groups ensuring that the questions were relevant, clear and understandable to a non-research audience.

### Step 3: Interim priority setting

To reduce the list of indicative questions before final prioritisation, a second survey was constructed to conduct an interim ranking. The survey was distributed to both steering groups, to private Facebook groups and to people from the first survey who agreed to participate in later steps. The second survey asked participants to score each indicative question from 1 to 10 based on their own knowledge and experience, where 1 = *Not important at all* and 10 = *Very important*. The order of the indicative questions was randomised for each respondent, aiming to reduce the risk of survey fatigue and selection bias, e.g. tending to have highest focus on the first and last questions. To ensure a fair representation in the prioritisation between the four groups, the five highest-scored indicative questions from each group were selected for the final priority setting workshop.

### Step 4: Final priority setting

An online workshop was held to establish the final top-10 list. People with prediabetes, people with type 2 diabetes, HCPs and researchers were invited to participate. The workshop used a nominal group technique and incorporated discussion activities in both small and large groups, which was facilitated by two individuals from the project group. Prior to the workshop, all participants received the list of indicative questions to consider their own prioritisation of the questions. The first part of the workshop included discussion of all questions aiming to identify the least important questions. Elimination of questions until ten questions were left was based on consensus from all participants. The second part focused on the importance of the remaining ten questions. To minimise the risk that one group or single individuals dominated the final decision, each participant anonymously submitted their own prioritised top-10 list. The individual ranked top-10 priorities were converted into a joint score for each question, where priority 1 = 10 points and priority 10 = 1 point. The joint score defined the ranking of the final top-10 list. In the event of a tie in joint scores, the research questions with most individual first priorities were ranked highest.

### International assessment

To ensure international relevance and impact, 20 key opinion leaders in prediabetes, obesity and/or diabetes identified from relevant international organisations (EASD, European Diabetes Epidemiology Group, European Association for the Study of Obesity and ADA) were invited to assess the international relevance of the top-10 list. The assessment was conducted through an online survey, where respondents were asked to rate the international relevance of each question on the same scale as used in the interim prioritisation. In addition, respondents were able to add suggestions for international research priorities in prediabetes that were not included in the identified list.

## Results

### Step 1: Gathering uncertainties from a national survey

The online survey was answered by 405 participants (see Table 1), made up of 68% patients, 9% relatives, 17% HCPs (of whom 33% were general practitioners, 54% nurses, 6% dietitians and 7% other) and 6% researchers. The group of people with type 2 diabetes had a diabetes duration between 0 and >20 years (0–1 years: 20%, 1–2 years: 18%, 2–5 years: 26%, 5–10 years: 11%, 10–20 years: 18%, >20 years: 7%) with the majority (64%) having less than 5 years duration. A total of 1142 answers were collected to: ‘What questions about prediabetes would you like to see answered by research?’, resulting in 2.8 submitted responses per participant. Furthermore, 210 (52%) of the participants agreed to take part in later steps of the PSP process. Visual inspection of the sensitivity analysis confirmed that the assumption of pooling the prediabetes and type 2 diabetes groups together was reasonable (ESM Figs 2, 3). The first PSP step took 7 months to complete, including setting up the PSP, with the survey being open for 3.5 months.

**Table 1:** Participant characteristics for steps one, three and four in the PSP process

Characteristic	Step 1: Survey questionnaire *n*=405 (%)	Step 3: Interim prioritisation *n*=115 (%)	Step 4: Final priority setting *n*=9 (%)
Participants			
Person with prediabetes	76 (18.8)	14 (12.2)	2 (22.2)
Person with T2D	201 (49.6)	51 (44.3)	1 (11.1)
Relative of a person with prediabetes or T2D	36 (8.9)	12 (10.4)	0 (0.0)
HCP	67 (16.5)	21 (18.3)	2 (22.2)
Researcher	25 (6.2)	16 (13.9)	3 (33.3)
Other	0 (0.0)	1 (0.9)	1 (11.1)
Gender			
Female	297 (73.3)	84 (73.0)	5 (55.6)
Male	105 (25.9)	31 (27.0)	4 (44.4)
Not known	3 (0.7)	0 (0.0)	0 (0.0)
Age			
18–25 years	2 (0.5)	0 (0.0)	0 (0.0)
26–40 years	66 (16.3)	22 (19.1)	2 (22.2)
41–55 years	96 (23.7)	30 (26.1)	4 (44.4)
56–70 years	203 (50.1)	54 (47.0)	3 (33.3)
≥71 years	38 (9.4)	9 (7.8)	0 (0.0)
Geography			
North Denmark Region	96 (23.7)	27 (23.5)	4 (44.4)
Central Denmark Region	82 (20.2)	19 (16.5)	0 (0.0)
Region of Southern Denmark	71 (17.5)	16 (13.9)	0 (0.0)
Region Zealand	59 (14.6)	14 (12.2)	1 (11.1)
Capital Region of Denmark	97 (24.0)	39 (33.9)	4 (44.4)
Highest educational level			
ISCED 1: Primary education	13 (3.2)	3 (2.6)	0 (0.0)
ISCED 2–4: Lower and upper secondary education and post-secondary non-tertiary education	50 (12.4)	9 (7.8)	1 (11.1)
ISCED 5: Short-cycle tertiary education	42 (10.4)	10 (8.7)	2 (22.2)
ISCED 6: Bachelor’s or equivalent level	199 (49.1)	58 (50.4)	0 (0.0)
ISCED 7–8: Master’s or equivalent level and Doctoral or equivalent level	99 (24.4)	34 (29.6)	6 (66.7)
Other	2 (0.5)	1 (0.9)	0 (0.0)

Categorisation of the educational levels follows the ISCED [24]

Data are presented as *n* (%)

ISCED, International Standard Classification of Education; T2D, type 2 diabetes

### Step 2: Organising uncertainties

**Fig. 2:**
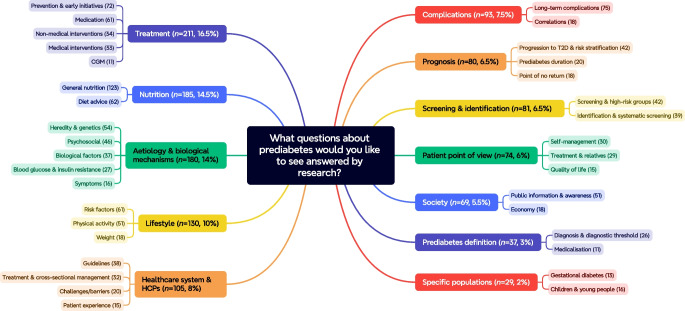
The 12 main themes and 35 sub-themes and how many times each main theme and sub-theme was mentioned in the collected uncertainties. T2D, type 2 diabetes

Initially, answers with more than one uncertainty in a single entry were separated, resulting in a total of 1429 gathered uncertainties from the first survey. Reviewing and interpretation of all submitted uncertainties resulted in 122 (9%) uncertainties excluded for being out-of-scope (*n*=9, 7%), missing information (*n*=11, 9%), incomplete row (*n*=98, 80%) or concerning a personal healthcare issue (*n*=4, 3%). The remaining 1307 uncertainties were organised into 68 initial themes. Subsequently, these initial themes were condensed into 12 main themes with 35 sub-themes (Fig. 2). Six sub-themes (including 33 uncertainties) were excluded due to low occurrence (<10 original uncertainties): fish oil/vitamins, funding of research, gut microbiota, pain, allergy and virus. The list including all 35 indicative questions can be found in ESM Table 1. Furthermore, information about how often participants within each of the different groups mentioned the different sub-themes can be found in ESM Fig. 4. The most frequently mentioned sub-theme for the groups of patients and relatives was *General nutrition* (16.5%). *Progression to type 2 diabetes & risk stratification* (15.1%) was most frequently mentioned for the group of researchers, whereas clinical *Guidelines* (17.9%) had the highest occurrence for the HCPs. The second PSP step was completed in 3 months.

### Step 3: Interim priority setting

**Fig. 3:**
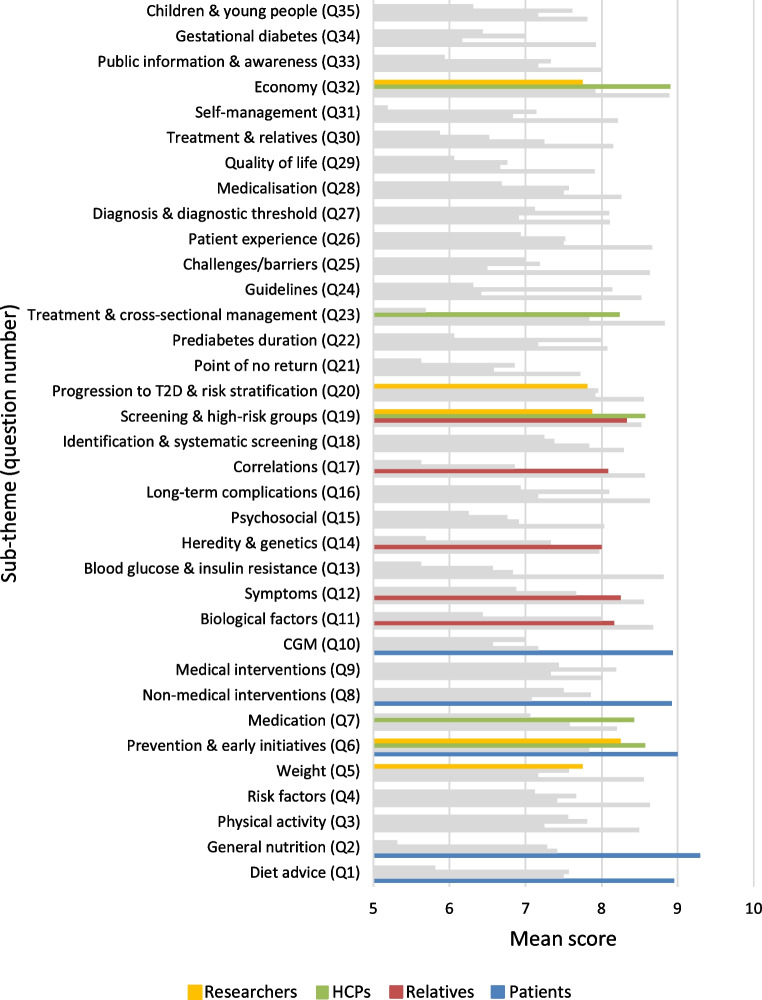
The mean score for each indicative question by each group from the interim prioritisation. The top-5 priorities for each group are highlighted in colours. T2D, type 2 diabetes

A total of 115 participants (see Table 1) took part in the interim prioritisation, which included scoring the 35 indicative questions. Of those who completed the prioritisation, 57% were patients, 10% relatives, 18% HCPs (9% general practitioners, 81% nurses and 10% other) and 14% researchers. The five highest ranked questions from each group resulted in 15 questions being selected for the final priority setting workshop (see Fig. 3). Preparation of the second survey, collection of responses and analysis of the results took 3 months.

### Step 4: Final priority setting

**Fig. 4:**
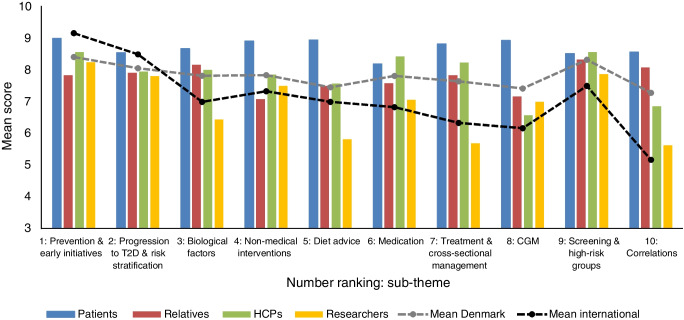
The mean score for each question on the identified top-10 list from each of the four stakeholder groups. Participants scored each indicative question from 1 to 10, where 1=*Not important at all* and 10=*Very important*. The mean from the Danish and international participants are shown by the dotted lines. T2D, type 2 diabetes

A total of 11 people (nine participants [see Table 1] and two facilitators) took part in the online workshop to establish the final top-10 list of the most important research priorities within prediabetes (see Table 2). The workshop took 3 h, however preparations started 3 months before. Five questions which were shortlisted for the final workshop but did not reach the top-10 list were questions regarding: *economy*, *weight*, *general nutrition*, *symptoms* and *heredity & genetics*. The mean interim score for each of the top-10 questions from each respondent group in Denmark are presented in Fig. 4. The full list of research questions can be found in ESM Table 1.

**Table 2:** Top-10 list showing the jointly highest prioritised research questions including the joint score from the final prioritisation workshop, and the mean scores from the Danish interim prioritisation survey and international assessment survey

Rank	Theme	Research question	Joint score	Mean score Denmark (international)
1	Prevention & early initiatives	What is the best prevention of diabetes, and will early prevention strategies reduce the number of people with T2D?	65	8.4 (9.2)
2	Progression to T2D & risk stratification	What characterises people with prediabetes progressing to T2D and can this knowledge be used to adjust the treatment to different groups?	59	8.1 (8.5)
3	Biological factors	How do biological factors such as blood pressure, cholesterol, hormones, weight, age and gender influence the development of prediabetes and the progression to T2D?	56	7.8 (7.0)
4	Non-medical interventions	Which non-medical interventions are most effective to prevent T2D and when should the intervention be initiated?	45	7.8 (7.3)
5	Diet advice	How can we target dietary advice and nutrition guidelines to people with prediabetes that are simple to follow and easy to maintain in the long term?	45	7.5 (7.0)
6	Medication	How do different types of pharmaceutical treatments (e.g. diabetes medication and weight loss medication) affect the development of prediabetes and how does it affect long-term complications?	43	7.8 (6.8)
7	Treatment & cross-sectional management	How can management of prediabetes be improved and can multidisciplinary collaboration among medical doctors, nurses, dietitians and other relevant stakeholders improve the prevention?	43	7.6 (6.3)
8	CGM	Can temporary use of CGM assist people with prediabetes to obtain a better understanding of how diet and physical activity affect the blood glucose levels and thereby be used to prevent the progression to T2D?	42	7.4 (6.2)
9	Screening & high-risk groups	Can screening be targeted to high-risk groups to optimise early identification of prediabetes and how can screening methods be implemented in clinical practice?	34	8.3 (7.5)
10	Correlations	Which relationships exist between other diseases and prediabetes, including autoimmune and endocrine diseases?	33	7.3 (5.2)

T2D, type 2 diabetes

### International assessment

Six people representing the UK, USA, Netherlands, Sweden and Denmark assessed the relevance of the top-10 research questions in an international context. Mean scores for each question from both the Danish participants and international group can be found in Fig. 4. The international group had a mean score across all ten questions of 7.1 compared with 7.8 in Denmark (patients = 8.7, relatives = 7.8, HCP= 7.9 and researchers = 6.9). No additional research questions were suggested by the international participants.

## Discussion

Our iterative and collaborative process between people with prediabetes, people with type 2 diabetes, relatives, HCPs, patient organisation and researchers identified the most important research priorities within prediabetes. The study provided 35 specific research questions and highlighted the highest prioritised questions on a top-10 list. Furthermore, an international assessment confirmed generalisability of the identified questions. Main topics of interest were research on prevention strategies (including both medical and non-medical interventions), risk factors, screening and personalised treatment. Additionally, differences in priorities were observed between patients, their relatives, researchers and HCPs. The results suggest a direction for the prediabetes research field based on a broad range of stakeholders, thus aiming to improve the relevance and clinical utility of future research within prediabetes.

The highest prioritised research question indicated a need to gain an overview of the effect of different prevention strategies and evaluation of whether these strategies will ultimately result in fewer people progressing to type 2 diabetes. Comparing the identified research priorities with the type 2 diabetes top-10 list, similar or overlapping themes were observed (e.g. *How do we identify people at high risk of type 2 diabetes and help to prevent the condition from developing?*) [14]. However, despite the close relationship between prediabetes and type 2 diabetes, research priorities were different for each research field highlighting the importance of individual priority settings. Aspects captured in the prediabetes top-10 list, which were not in the type 2 diabetes top-10 list, included uncertainties regarding effect of pharmaceutical treatments, use of continuous glucose monitoring (CGM) as a learning tool, management of prediabetes with a focus on multidisciplinary collaboration, and understanding of biological factors affecting progression from prediabetes to type 2 diabetes.

To understand the suggested direction for future prediabetes research, it is essential to compare our findings with the existing research field. A study explored the global research trends in prediabetes between 2013 and 2022 and identified five research clusters [1]. Sub-themes within these clusters are closely aligned with the sub-themes identified in our study (e.g. risk, prevalence, complications, pathogenesis, risk factors, lifestyle intervention, primary care, impaired glucose tolerance and metabolic syndrome with pregnancy, cardiovascular risk, dyslipidaemia); however, our study provided more detailed insights into these topics. Additionally, our suggested top-10 list differs by emphasising use of CGM, personalised treatment and pharmaceutical interventions. Further, patient-centric research questions (e.g. questions related to patient experience and quality of life), which were observed outside the top-10 list, are currently not prioritised in the research field.

The international assessment of the top-10 list showed a similar trend between the Danish and international scores, thus confirming generalisability of the identified top-10 list. The slightly lower mean for the international group (7.1) across all ten questions compared with Denmark (7.8) could be explained by individuals with prediabetes and type 2 diabetes not being included in the international group. The international participants were assessed to be similar to the researcher group in Denmark based on their educational background and engagement in relevant international organisations focusing on research. In the Danish survey, the patient group had the highest mean score across all ten questions compared with the three other groups. Therefore, although the study was based on participants and research questions in Denmark, the results indicate international generalisability.

In both surveys each stakeholder group provided different views and priorities. When gathering uncertainties, most frequently mentioned topics from patients were *general nutrition*, *diet advice* and *public information & awareness*, whereas the most frequently mentioned topics from HCPs were within clinical *guidelines*, *medication* and *medical interventions*. From the interim prioritisation, four out of five questions on the patients’ and relatives’ top-5 list were only prioritised by these two groups. HCPs and researchers agreed on three out of five questions in their top-5 lists, indicating that these two groups were more similar in their priorities. *Diet*, *non-medical interventions* and use of *CGM* seemed to be the most important topics for patients, whereas *biological factors*, *aetiology* and *symptoms* were most important to relatives. *Economy*, *screening & high-risk groups*, and *prevention & early initiatives* were important to both HCPs and researchers. *Medication* and *cross-sectorial management* were prioritised only by HCPs, whereas *weight* and *progression to type 2 diabetes* were prioritised only by researchers. Differences in priorities between groups were also observed in the PSP within type 2 diabetes, indicating the importance of involving several different stakeholders to capture a comprehensive range of perspectives. Furthermore, these findings align with literature indicating that non-drug treatment in general is preferred by patients and that research on drugs is prioritised by researchers [16].

It should be noted that the top-10 list is not definitive and that questions outside the top-10 are also important to investigate. It is well known that the methodology applied at the workshop contains subjectivity in participants’ prioritisation, thus recruitment of another set of people could give different results. However, ranking similarities between interim and final prioritisation enhanced the reliability of the final list. The highest prioritised question on the top-10 list regarding *prevention & early initiatives* was also the highest jointly ranked question from the interim prioritisation. Similarly, the second and third ranked questions on the final top-10 list were jointly ranked as number four and five from the interim prioritisation, respectively. At the same time, however, the jointly second highest ranked question (*economy*) and the patients’ highest ranked question (*general nutrition*) in the interim prioritisation were not included in the final top-10 list. These changes in priorities between the interim and final prioritisation have been observed in previous PSPs, indicating that sharing and discussing different perspectives among stakeholders during the final priority setting can affect the individual’s priorities [14, 20, 25, 26]. Decision after group discussion compared with individual decision has been shown in the literature to reduce bias and to help use information more effectively, so each individual’s decisions will be based on a broader set of experiences [27]. Thus, facilitation of group discussions at the final prioritisation improved the quality and reduced potential biases.

Reformulation and rearrangement of some questions were suggested by participants at the final workshop. In general, it was suggested to focus explicitly on people with prediabetes at highest risk for progression to type 2 diabetes instead of all people with prediabetes, since a lot of people with prediabetes will return to normoglycaemia [11]. In addition to this, the importance of discussing the term ‘prediabetes’ was mentioned. Existing literature indicates contradictory findings regarding potential implications if people are ‘diagnosed’ with prediabetes [10, 28, 29]. This topic was captured in the question regarding medicalisation: *Should prediabetes be managed at all? And if so, should it be called ‘prediabetes’, and how can stigmatisation and over-treatment of this group be avoided?* Thus, even though this question was not shortlisted for the final workshop, and therefore not in the top-10, the topic seemed crucial to consider and discuss in future research.

Key strengths of this study included the systematic and transparent approach applied through the entire PSP process and the equal involvement and weighting of perspectives from patients, relatives, HCPs and researchers. To ensure that a broad representative range of perspectives were captured, an online survey was distributed through several platforms to all geographic areas of Denmark. The study succeeded in recruiting an acceptable number of people to each group, representing diversity in age, geography and educational levels. However, a disparity between the representation of women (~73%) and men (~26%) were observed in both survey questionnaires. Despite attempts to target Facebook advertisements specifically to men, we were not able to recruit an equal number of women and men, which could have introduced some bias to the results. Furthermore, the educational levels tended to be higher than expected in the general population of people with prediabetes, thus perspectives of individuals with lower educational backgrounds may not be captured in our analysis [30]. Therefore, due to the challenging nature of identifying and thereby recruiting people with prediabetes, it is unknown whether the sample of people with prediabetes included in this study represent the broader population of people with prediabetes. We relied on participants to self-report their status as either having prediabetes or type 2 diabetes, because it was not feasible to conduct a confirmatory blood test due to time and funding. Moreover, a sensitivity analysis showed similar patterns in the responses originating from people with prediabetes and type 2 diabetes. Additionally, information on ethnicity was not collected about the participants; thus, it was not known whether the sample in this study represents priorities from different ethnic backgrounds. Future studies could benefit from collecting information on ethnicity and providing surveys in both Danish and English, thus enabling collection of uncertainties and prioritisation from people with different ethnicities and nationalities [31, 32]. Lastly, there might be a risk of missing perspectives on what is important in general when focusing only on uncertainties. However, we deviated from the traditional PSP methodology by not assessing uncertainties as ‘true uncertainties’, which possibly allowed us to capture a wider range of important perspectives.

To maximise awareness and the scientific impact of the identified research questions, future work should focus on a post-PSP strategy addressing how researchers, funders and the healthcare industry should turn the identified research questions into action [33]. Integrating these shared priorities when setting the scope for new research in prediabetes will improve relevance and enhance the possibility that research will directly impact individuals with prediabetes and HCPs.

### Conclusion

This study identified a top-10 list of research priorities highlighting the most important shared priorities from individuals with prediabetes, HCPs and relevant stakeholders in prediabetes. The research questions covered several aspects of prediabetes encompassing prevention strategies including both medical and non-medical interventions, diet advice, use of CGMs, risk factors, screening and personalised treatment. In general, individuals with prediabetes or type 2 diabetes and their relatives had different priorities from researchers and HCPs. Based on the results, academia, funders and the healthcare industry should target their research within prediabetes to the specific needs of patients and HCPs, aiming to enhance the management and health of people living with prediabetes.

## Supplementary Information

Below is the link to the electronic supplementary material.

ESM (PDF 1705 KB)

## Data Availability

Individual participant data have been provided to this study on the understanding that it would be confidential and used only for the purpose of analysing research uncertainties and describing participant demographics for the specific study. Thus, data collected for the study will not be released to any third party.

## Abbreviations

CGM: Continuous glucose monitoring
HCP: Healthcare professional
IEC: International Expert Committee
JLA: James Lind Alliance
PSP: Priority setting partnership

## Authors’ relationships and activities

AKA is a Novo Nordisk employee and has received a PhD grant from the DDEA (grant number NNF22SA0079901) and the Danish Data Science Academy (grant number NNF21SA0069429), funded by the Novo Nordisk Foundation. KF is a Novo Nordisk employee and holds Novo Nordisk shares. Additionally, KF is a member of the DDEA Advisory Board and Board Committee on Strategic Partnerships. DV is a Novo Nordisk employee and holds Novo Nordisk shares. BJS is a Novo Nordisk employee and holds Novo Nordisk shares. MHJ is a Novo Nordisk employee and holds Novo Nordisk shares. The remaining authors declare that there are no relationships or activities that might bias, or be perceived to bias, their work.

## References

[1] Wang G, Chen Y, Liu X, Ma S, Jiang M (2024) Global research trends in prediabetes over the past decade: Bibliometric and visualized analysis. Medicine (Baltimore) 103(3):e36857. 10.1097/MD.0000000000036857 38241546 10.1097/MD.0000000000036857 PMC10798732

[2] Rooney MR, Fang M, Ogurtsova K et al (2023) Global prevalence of prediabetes. Diabetes Care 46(7):1388–1394. 10.2337/dc22-2376 37196350 10.2337/dc22-2376 PMC10442190

[3] IEC (2009) International expert committee report on the role of the A1C assay in the diagnosis of diabetes. Diabetes Care 32(7):1327–1334. 10.2337/dc09-9033 19502545 10.2337/dc09-9033 PMC2699715

[4] WHO (2011) Use of glycated haemoglobin (HbA1c) in the diagnosis of diabetes mellitus: abbreviated report of a WHO consultation. World Health Organization, Geneva 26158184

[5] Færch K, Vistisen D, Johansen NB, Jørgensen ME (2014) Cardiovascular risk stratification and management in pre-diabetes. Curr Diab Rep 14(6):493. 10.1007/s11892-014-0493-1 24743942 10.1007/s11892-014-0493-1

[6] Kim SH (2024) Reframing prediabetes: a call for better risk stratification and intervention. J Int Med 295(6):735–747. 10.1111/joim.13786 10.1111/joim.13786 38606904

[7] American Diabetes Association Professional Practice Committee (2023) 2. Diagnosis and classification of diabetes: standards of care in diabetes—2024. Diabetes Care 47(Supplement_1):S20–S42. 10.2337/dc24-S002 10.2337/dc24-S002 PMC10725812 38078589

[8] Echouffo-Tcheugui JB, Selvin E (2021) Pre-diabetes and what it means: the epidemiological evidence. Annu Rev Public Health 42:59. 10.1146/annurev-publhealth-090419-102644 33355476 10.1146/annurev-publhealth-090419-102644 PMC8026645

[9] Centers for Disease Control and Prevention (2024) National Diabetes Statistics Report. Diabetes. https://www.cdc.gov/diabetes/php/data-research/index.html. Accessed 24 Jun 2024

[10] Richter B, Hemmingsen B, Metzendorf M, Takwoingi Y (2018) Development of type 2 diabetes mellitus in people with intermediate hyperglycaemia. Cochrane Database Syst Rev 10:CD012661. 10.1002/14651858.CD012661.pub2 30371961 10.1002/14651858.CD012661.pub2 PMC6516891

[11] Tabák AG, Herder C, Rathmann W, Brunner EJ, Kivimäki M (2012) Prediabetes: A high-risk state for developing diabetes. Lancet 379(9833):2279–2290. 10.1016/S0140-6736(12)60283-9 22683128 10.1016/S0140-6736(12)60283-9 PMC3891203

[12] Echouffo-Tcheugui JB, Perreault L, Ji L, Dagogo-Jack S (2023) Diagnosis and management of prediabetes: a review. JAMA 329(14):1206–1216. 10.1001/jama.2023.4063 37039787 10.1001/jama.2023.4063

[13] Gadsby R, Snow R, Daly AC et al (2012) Setting research priorities for type 1 diabetes. Diabetic Med 29(10):1321–1326. 10.1111/j.1464-5491.2012.03755.x 22823450 10.1111/j.1464-5491.2012.03755.x

[14] Finer S, Robb P, Cowan K, Daly A, Shah K, Farmer A (2018) Setting the top 10 research priorities to improve the health of people with Type 2 diabetes: a Diabetes UK–James Lind Alliance Priority Setting Partnership. Diabet Med 35(7):862–870. 10.1111/dme.13613 29485717 10.1111/dme.13613 PMC6032840

[15] Badrick E, Cresswell K, Ellis P et al (2019) Top ten research priorities for detecting cancer early. Lancet Public Health 4(11):e551. 10.1016/S2468-2667(19)30185-9 31562068 10.1016/S2468-2667(19)30185-9

[16] Crowe S, Fenton M, Hall M, Cowan K, Chalmers I (2015) Patients’, clinicians’ and the research communities’ priorities for treatment research: there is an important mismatch. Res Involv Engagem 1:2. 10.1186/s40900-015-0003-x 29062491 10.1186/s40900-015-0003-x PMC5598091

[17] Skovlund PC, Nielsen BK, Thaysen HV et al (2020) The impact of patient involvement in research: a case study of the planning, conduct and dissemination of a clinical, controlled trial. Res Involv Engagem 6:43. 10.1186/s40900-020-00214-5 32699648 10.1186/s40900-020-00214-5 PMC7370448

[18] South A, Hanley B, Gafos M et al (2016) Models and impact of patient and public involvement in studies carried out by the Medical Research Council Clinical Trials Unit at University College London: findings from ten case studies. Trials 17:376. 10.1186/s13063-016-1488-9 27473060 10.1186/s13063-016-1488-9 PMC4966697

[19] Jagosh J, Macaulay AC, Pluye P et al (2012) Uncovering the benefits of participatory research: implications of a realist review for health research and practice. Milbank Q 90(2):311–346. 10.1111/j.1468-0009.2012.00665.x 22709390 10.1111/j.1468-0009.2012.00665.x PMC3460206

[20] NIHR (2021) The James Lind Alliance Guidebook, Version 10. https://www.jla.nihr.ac.uk/jla-guidebook/downloads/JLA-Guidebook-Version-10-March-2021.pdf. Accessed 22 Feb 2024

[21] Harris PA, Taylor R, Minor BL et al (2019) The REDCap consortium: Building an international community of software platform partners. J Biomed Inform 95:103208. 10.1016/j.jbi.2019.103208 31078660 10.1016/j.jbi.2019.103208 PMC7254481

[22] Quist JS, Pedersen HE, Jensen MM et al (2024) Effects of 3 months of 10-h per-day time-restricted eating and 3 months of follow-up on bodyweight and cardiometabolic health in Danish individuals at high risk of type 2 diabetes: the RESET single-centre, parallel, superiority, open-label, randomised controlled trial. Lancet Healthy Longev 5(5):e314–e325. 10.1016/S2666-7568(24)00028-X 38588687 10.1016/S2666-7568(24)00028-X

[23] Jalking L, Launbo NP, Jensen MM et al (2025) Effects of exercise and exercise timing on energy intake and appetite control in Danish individuals with overweight or obesity with and without type 2 diabetes: a protocol for a randomised controlled cross-over trial. BMJ Open 15(2):e092683. 10.1136/bmjopen-2024-092683 39915022 10.1136/bmjopen-2024-092683 PMC11800291

[24] UNESCO (2012) International Standard Classification of Education - ISCED 2011. UNESCO Institute for Statistics, Canada

[25] Ayman G, Strachan JA, McLennan N et al (2021) The top 10 research priorities in diabetes and pregnancy according to women, support networks and healthcare professionals. Diabet Med 38(8):e14588. 10.1111/dme.14588 33949704 10.1111/dme.14588 PMC8359941

[26] Taylor CJ, Huntley AL, Burden J et al (2020) Research priorities in advanced heart failure: James Lind alliance priority setting partnership. Open Heart 7(1):e001258. 10.1136/openhrt-2020-001258 32606070 10.1136/openhrt-2020-001258 PMC7328807

[27] Bang D, Frith CD (2017) Making better decisions in groups. R Soc Open Sci 4(8):170193. 10.1098/rsos.170193 28878973 10.1098/rsos.170193 PMC5579088

[28] Chakkalakal RJ, Galaviz KI, Thirunavukkarasu S, Shah MK, Narayan KMV (2024) Test and treat for prediabetes: a review of the health effects of prediabetes and the role of screening and prevention. Annu Rev Public Health 45:151–167. 10.1146/annurev-publhealth-060222-023417 38109519 10.1146/annurev-publhealth-060222-023417

[29] Roper KL, Thomas AR, Hieronymus L, Brock A, Keck J (2019) Patient and clinician perceptions of prediabetes: a mixed-methods primary care study. Diabetes Educ 45(3):302–314. 10.1177/0145721719845347 31018784 10.1177/0145721719845347

[30] Nicolaisen SK, Pedersen L, Witte DR, Sørensen HT, Thomsen RW (2023) HbA1c-defined prediabetes and progression to type 2 diabetes in Denmark: a population-based study based on routine clinical care laboratory data. Diabetes Res Clin Pract 203:110829. 10.1016/j.diabres.2023.110829 37451628 10.1016/j.diabres.2023.110829

[31] Iqbal H, West J, Haith-Cooper M, McEachan RRC (2021) A systematic review to identify research priority setting in Black and minority ethnic health and evaluate their processes. PLOS ONE 16(5):e0251685. 10.1371/journal.pone.0251685 34048459 10.1371/journal.pone.0251685 PMC8162667

[32] Dawson S, Campbell SM, Giles SJ, Morris RL, Cheraghi-Sohi S (2018) Black and minority ethnic group involvement in health and social care research: a systematic review. Health Expect 21(1):3–22. 10.1111/hex.12597 28812330 10.1111/hex.12597 PMC5750731

[33] Staley K, Crowe S, Crocker JC, Madden M, Greenhalgh T (2020) What happens after James Lind Alliance Priority Setting Partnerships? A qualitative study of contexts, processes and impacts. Res Involv Engagem 6:41. 10.1186/s40900-020-00210-9 32670611 10.1186/s40900-020-00210-9 PMC7353437

