# Supplementary material for: What are the most important research questions within prediabetes? A priority setting partnership in collaboration with patients, healthcare professionals and researchers
Source: Diabetologia. 2025 Aug 4;68(10):2156–67. doi: 10.1007/s00125-025-06505-4 (PMC12423201; doi:10.1007/s00125-025-06505-4)
Supplement: Supplementary file 1 — ESM (PDF 1705 KB) [file 125_2025_6505_MOESM1_ESM.pdf]

Electronic supplementary material (ESM)

ESM Table 1: List of the 35 indicative questions including main theme, sub-theme, number of uncertainties and explanation for each indicative question.

**Main theme**	**Sub-theme**	**Indicative question (uncertainty)**	**Number of uncertainties (%)**		**Explanation**
**Nutrition**	Diet advice	How can we target dietary advice and nutrition guidelines to people with prediabetes that are simple to follow and easy to maintain in the long term?	Total<br>Patients<br>Relatives<br>HCPs<br>Researchers	62<br>56 (90.3)<br>2 (3.2)<br>2 (3.2)<br>2 (3.2)	This question focuses on how dietary advice and guidance can be simplified to make it easy to implement in daily life and maintain in the long term. It also addresses the need for guidance to be specifically targeted to individuals with prediabetes, rather than being combined with Type 2 Diabetes. Furthermore, a specific focus on the National Health Authority and the 'food pyramid' was observed.
	General nutrition	What diet is most effective for preventing diabetes? And what are the pros and cons of a low-carbohydrate diet?	Total<br>Patients<br>Relatives<br>HCPs<br>Researchers	123<br>108 (87.8)<br>12 (9.8)<br>3 (2.4)<br>0 (0.0)	This question concerns whether the progression to Type 2 Diabetes can be prevented through diet. Furthermore, how various specific types of diets (e.g. LCHF (low carb high fat), fasting, vegetarian) affect the prediabetes condition. For instance, the effect of a LCHF diet in respect to elevated cholesterol levels and the risk of cardiovascular diseases.
**Lifestyle**	Physical activity	How does exercise affect the development of prediabetes? Including, how much exercise is necessary to see a preventive effect and what type of exercise is most effective?	Total<br>Patients<br>Relatives<br>HCPs<br>Researchers	51<br>34 (66.7)<br>7 (13.7)<br>7 (13.7)<br>3 (5.9)	Focuses on the effect of exercise and physical activity in the prevention and treatment of prediabetes. Discusses whether some types of exercise are more effective than others and how much exercise is necessary to achieve beneficial effects.

	Risk factors	How do lifestyle factors (diet, exercise, smoking, alcohol) affect the development of prediabetes, and which of these factors are most important for the progression to T2D?	Total 61<br>Patients 34 (55.7)<br>Relatives 4 (6.6)<br>HCPs 17 (27.9)<br>Researchers 6 (9.8)	Focuses on the impact of different lifestyle factors and which changes to an individuals' lifestyle are most important to prevent Type 2 Diabetes if prioritization is necessary.
	Weight	How does body weight and a potential weight loss affect prediabetes, and is weight loss or exercise most important for preventing T2D?	Total 18<br>Patients 14 (77.8)<br>Relatives 1 (5.6)<br>HCPs 3 (16.7)<br>Researchers 0 (0.0)	The question focusses on the importance of body weight and weight loss in relation to prediabetes and Type 2 Diabetes. The question originates from uncertainties about how much weight loss is necessary or sufficient to reduce the risk of prediabetes/Type 2 Diabetes. Additionally, it covers whether weight loss alone can prevent the condition and whether medical treatments (such as GLP-1) can have an effect. Uncertainties about weight loss are often combined with topics as diet and exercise.
**Treatment**	Prevention & Early initiatives	What is the best prevention of diabetes, and will early prevention strategies reduce the number of people with T2D?	Total 72<br>Patients 41 (56.9)<br>Relatives 6 (8.3)<br>HCPs 14 (19.4)<br>Researchers 11 (15.3)	Focuses more generally on treatment and early prevention, and whether early prevention strategies will ultimately result in less people being diagnosed with Type 2 Diabetes.
	Medication	How do different types of pharmaceutical treatments (e.g. diabetes medication and weight loss medication) affect the development of prediabetes and how does it affect long-term complications?	Total 61<br>Patients 32 (52.5)<br>Relatives 5 (8.2)<br>HCPs 23 (37.7)<br>Researchers 1 (1.6)	Addresses various aspects of medical treatment. Many original uncertainties focused on the effectiveness of different medications (GLP-1, SGLT2, Semaglutide, Metformin, Ozempic, and Wegovy) – specifically regarding whether these treatments can prevent or delay the development of diabetes, for whom and when the treatment should be initiated, and which medication is most effective in preventing long-term complications. Additionally, there is a focus on medication in combination with diet/exercise – for example, whether lifestyle changes alone are sufficient, or if medical treatment should be used as a supplement.

	Non-medical interventions	Which non-medical interventions are most effective to prevent T2D and when should the intervention be initiated?	Total 34<br>Patients 10 (29.4)<br>Relatives 4 (11.8)<br>HCPs 12 (35.3)<br>Researchers 8 (23.5)	Addresses whether some non-medical interventions are more effective to prevent Type 2 Diabetes. Furthermore, which interventions should be suggested to individuals with prediabetes and when should it be initiated.
	Medical interventions	When should medical treatment be initiated to achieve the best effect, and which patients would benefit the most from medication rather than lifestyle interventions alone?	Total 33<br>Patients 4 (12.1)<br>Relatives 2 (6.1)<br>HCPs 22 (66.7)<br>Researchers 5 (15.2)	Focusing on medical interventions for people with prediabetes. Which type of medical treatment is most effective and at what time (e.g. HbA1c level) should it be initiated. Additionally, there is a specific focus on personalized treatment.
	CGM	Can temporarily use of a continuous glucose monitoring (CGM) assist people with prediabetes to obtain a better understanding of how diet and physical activity affect the blood glucose levels and thereby be used to prevent the progression to T2D?	Total 11<br>Patients 6 (54.5)<br>Relatives 2 (18.2)<br>HCPs 0 (0.0)<br>Researchers 3 (27.3)	Focuses on whether temporary use of continuous glucose monitors (e.g. 14 days, 2 months, 6 months) can help people with prediabetes to obtain a better understanding of how blood glucose is affected by factors such as diet, exercise, and alcohol, thus being used as a preventive learning tool.
**Etiology & Biological Mechanisms**	Biological factors	How do biological factors such as blood pressure, cholesterol, hormones, weight, age, and gender influence the development of prediabetes and the progression to T2D?	Total 37<br>Patients 25 (67.6)<br>Relatives 3 (8.1)<br>HCPs 3 (8.1)<br>Researchers 6 (16.2)	The question originates from uncertainties regarding various biological factors. Mainly focusing on which effect these factors have for the development of prediabetes and the progression to Type 2 Diabetes.
	Symptoms	Are there early signs or symptoms that can be used to alert people about the presence of prediabetes?	Total 16<br>Patients 8 (50.0)<br>Relatives 5 (31.3)<br>HCPs 3 (18.8)<br>Researchers 0 (0.0)	The questions focus on whether there exists any early signs or symptoms, that can be used to make people aware that they are in the prediabetes range.
	Blood glucose & Insulin resistance	What impact do fluctuations in blood glucose and insulin resistance have on the development of prediabetes?	Total 27<br>Patients 20 (74.1)<br>Relatives 5 (18.5)<br>HCPs 1 (3.7)<br>Researchers 1 (3.7)	The questions focus specifically on uncertainties regarding blood glucose fluctuations an insulin resistance. E.g. whether a slightly increased but stable blood glucose level is better/worse than a lower mean blood glucose level with high daily spikes. And how does insulin resistance affect the prediabetes condition.

	Heredity & Genetics	How does heredity and genetics influence the risk of prediabetes?	Total<br>Patients<br>Relatives<br>HCPs<br>Researchers	54<br>36 (66.7)<br>7 (13.0)<br>8 (14.8)<br>3 (5.6)	Focusing on the impact of heredity and genetics. E.g. whether some people are in higher risk of prediabetes due to their genes.
	Psychosocial	What influence do psychosocial factors such as stress, anxiety, grief, sleep, finances, and level of education have on the development of prediabetes?	Total<br>Patients<br>Relatives<br>HCPs<br>Researchers	46<br>32 (69.6)<br>7 (15.2)<br>3 (6.5)<br>4 (8.7)	Addresses how psychosocial factors can affect the development of prediabetes. For example, if/how stress, anxiety, or depression can trigger prediabetes?
**Complications**	Long-term complications	What are the long-term health consequences and complications of prediabetes? Including, what is the risk and when do these consequences occur?	Total<br>Patients<br>Relatives<br>HCPs<br>Researchers	75<br>40 (53.3)<br>10 (13.3)<br>15 (20.0)<br>10 (13.3)	The question focuses on complications and comorbidities related to prediabetes. Specific diseases and organs mentioned were neuropathy, cardiovascular diseases, eyes, kidneys, teeth and pancreas.
	Correlations	Which relationships exist between other diseases and prediabetes, including autoimmune and endocrine diseases?	Total<br>Patients<br>Relatives<br>HCPs<br>Researchers	18<br>15 (83.3)<br>2 (11.1)<br>0 (0.0)<br>1 (5.6)	Addresses the relationship between other diseases and prediabetes. Specifically, the following diseases/conditions were mentioned: Hashimotos, autoimmune disorders, PCOS, slow metabolism, hormone diseases Morbus Bechterew, and paradentosis
**Screening & Identification**	Identification & Systematic screening	What is the best way to identify people with prediabetes? And what are the advantages and disadvantages of systematic screening for prediabetes in the population?	Total<br>Patients<br>Relatives<br>HCPs<br>Researchers	39<br>13 (33.3)<br>2 (5.1)<br>12 (30.8)<br>12 (30.8)	Focus on how to find people with prediabetes. A specific focus is on systematic screening tools and the pros and cons if using these tools to identify people with prediabetes.
	Screening & high-risk groups	Can screening be targeted to high-risk groups to optimize early identification of prediabetes? And how can screening methods be implemented in clinical practice?	Total<br>Patients<br>Relatives<br>HCPs<br>Researchers	42<br>24 (57.1)<br>0 (0.0)<br>11 (26.2)<br>7 (16.7)	Focus on whether screening methods can be targeted to high-risk groups and whether screening methods can be implemented in clinical practice (e.g. at the general practitioner)
**Prognosis**	Progression to T2D & risk stratification	What characterizes people with prediabetes progressing to T2D? And can this knowledge be used to adjust the treatment to different groups?	Total<br>Patients<br>Relatives<br>HCPs<br>Researchers	42<br>14 (33.3)<br>2 (4.8)<br>12 (28.6)<br>14 (33.3)	This question addresses the progression to Type 2 Diabetes, including how and whether it is possible to predict which individuals with prediabetes, who will progress to Type 2 Diabetes. Furthermore, the question regards what characterizes people

				with prediabetes who develop Type 2 Diabetes compared to those with prediabetes who return to normoglycemia. Additionally, whether algorithms can be used to identify patients at high risk of Type 2 Diabetes to determine which patients would benefit most from treatment.
	Point of no return	Is there a point of no return, where it is not possible to avoid progression to T2D?	Total 18<br>Patients 12 (66.7)<br>Relatives 2 (11.1)<br>HCPs 2 (11.1)<br>Researchers 2 (11.1)	The questions focus on whether it is possible to reverse the condition before it develops to Type 2 Diabetes. Specifically, if there is a point at which it is no longer possible to avoid developing Type 2 Diabetes.
	Prediabetes duration	How long time does a person typically remain in the prediabetes stage, and how does the duration affect the risk of complications?	Total 20<br>Patients 4 (20.0)<br>Relatives 3 (15.0)<br>HCPs 8 (40.0)<br>Researchers 5 (25.0)	Focusing on the duration of prediabetes. What is the typical duration of prediabetes (e.g., how long time is a person typically between HbA1c from 42 to 48). Furthermore, uncertainties focus on how different factors affect how quickly the progression occurs, what risks are associated with prediabetes at different durations, and how does the risk of complications change if one returns to normal levels.
**Healthcare system & HCPs**	Treatment & Cross-sectional management	How can management of prediabetes be improved? And can multidisciplinary collaboration among medical doctors, nurses, dieticians, and other relevant stakeholders improve the prevention?	Total 32<br>Patients 15 (46.9)<br>Relatives 3 (9.4)<br>HCPs 8 (25.0)<br>Researchers 6 (18.8)	This question focusses on the management of prediabetes. A more holistic approach and multidisciplinary collaboration is suggested in the submitted uncertainties.
	Guidelines	How can guidelines assist healthcare professionals in managing prediabetes?	Total 38<br>Patients 5 (13.2)<br>Relatives 2 (5.3)<br>HCPs 29 (76.3)<br>Researchers 2 (5.3)	Focusing on how guidelines on prediabetes can assist the healthcare professionals. The original uncertainties originate mainly from HCPs who asks for more/better guidelines to assist the HCP in deciding when treatment should be initiated and which people with prediabetes are most important to focus on.

	Challenges/barriers	What challenges and barriers are present among general practitioners in managing prediabetes in primary care?	Total 20<br>Patients 16 (80.0)<br>Relatives 1 (5.0)<br>HCPs 2 (10.0)<br>Researchers 1 (5.0)	The question focuses on the challenges and barriers that exist among general practitioners and in primary care. E.g. whether the general practitioners need more specific knowledge to manage prediabetes and uncertainties related to the fact that prediabetes is not defined as a 'disease' in Denmark.
	Patient experience	How does lack of attention to prediabetes by general practitioners affect patients' perception of the possibility to receive help during the prediabetes stage?	Total 15<br>Patients 14 (93.3)<br>Relatives 1 (6.7)<br>HCPs 0 (0.0)<br>Researchers 0 (0.0)	The question focuses on uncertainties from the patients' point of view. For example, some patients feels that prediabetes is just a waiting period, where it is difficult to get any help to manage the condition.
**Prediabetes definition**	Diagnosis & diagnostic threshold	How can a clear definition of prediabetes be established, and should the boundary for T2D be moved down into the prediabetes range?	Total 26<br>Patients 17 (65.4)<br>Relatives 2 (7.7)<br>HCPs 5 (19.2)<br>Researchers 2 (7.7)	Addresses whether the prediabetes definition should be changed and whether the lower threshold for Type 2 Diabetes should be moved down into the prediabetes range.
	Medicalisation	Should prediabetes be managed at all? And if so, should it be called 'prediabetes', and how can stigmatization and over-treatment of this group be avoided?	Total 11<br>Patients 3 (27.3)<br>Relatives 0 (0.0)<br>HCPs 3 (27.3)<br>Researchers 5 (45.5)	This question focusing on the term 'prediabetes'. This includes topics as stigmatization and over-treatment.
**Patient point of view**	Quality of life	How does prediabetes affect the daily life, mental well-being, and quality of life, and what can help manage prediabetes in daily life?	Total 15<br>Patients 7 (46.7)<br>Relatives 5 (33.3)<br>HCPs 0 (0.0)<br>Researchers 3 (20.0)	Focusing on how prediabetes affects the daily life and mental well-being.
	Treatment & relatives	How do individuals with prediabetes feel they are best supported in managing prediabetes? And can relatives be involved to improve prevention and maintenance of lifestyle changes?	Total 29<br>Patients 16 (55.2)<br>Relatives 5 (17.2)<br>HCPs 2 (6.9)<br>Researchers 6 (20.7)	This question is about the needs and preferences from a patient point of view and whether relatives can be involved to improve the effect of non-medical interventions.

	Self-management	How can people with prediabetes be guided/educated about the condition and potential interventions? And can an online forum/social community for people with prediabetes support this process?	Total<br>Patients<br>Relatives<br>HCPs<br>Researchers	30<br>22 (73.3)<br>5 (16.7)<br>1 (3.3)<br>2 (6.7)	Addresses how people with prediabetes can be assisted in managing their condition. Specific topics mentioned are help to lifestyle changes, patient education, use of CGM, online forum/social community for people with prediabetes.
**Society**	Economy	Is it beneficial for the society to manage prediabetes?	Total<br>Patients<br>Relatives<br>HCPs<br>Researchers	18<br>6 (33.3)<br>1 (5.6)<br>5 (27.8)<br>6 (33.3)	This question focusses on the economically aspects of prediabetes seen from a society point of view. Specifically, different cost-benefit analyses and whether it will be beneficial for the society to manage prediabetes.
	Public information & awareness	How can the society best inform and educate the population about prediabetes? And how can the society influence people with prediabetes towards a healthier lifestyle through taxes, fees, guidance, and dietary advice?	Total<br>Patients<br>Relatives<br>HCPs<br>Researchers	51<br>44 (86.3)<br>5 (9.8)<br>2 (3.9)<br>0 (0.0)	Focusing on how the society can be better to inform and educate the population about prediabetes.
**Specific population**	Gestational diabetes	How can education and follow-up be implemented to prevent prediabetes and T2D in women who have had gestational diabetes?	Total<br>Patients<br>Relatives<br>HCPs<br>Researchers	13<br>10 (76.9)<br>0 (0.0)<br>3 (23.1)<br>0 (0.0)	This question focusses specifically on the relation between gestational diabetes and prediabetes. E.g. if preventive initiatives should be targeted specifically for this group.
	Children & Young People	How can initiatives in primary schools prevent prediabetes in children and adolescents? And what behavioural patterns and characteristics are present in children and adolescents who are at highest risk of developing prediabetes?	Total<br>Patients<br>Relatives<br>HCPs<br>Researchers	16<br>7 (43.8)<br>4 (25.0)<br>1 (6.3)<br>4 (25.0)	Addressing whether early preventive initiatives should be implemented in primary schools. Additionally, there is a focus on which patterns and characteristics are present in children at high risk of prediabetes.

ESM Figure 1

Om dig

Hvilke af følgende udsagn passer bedst på dig?<br><br>Hvis flere udsagn passer på dig, kan du vælge andet og uddybe i kommentarfeltet.	○ Person på +18 år med prædiabetes<br>○ Person på +18 år med type 2-diabetes<br>○ Pårørende til en voksen med prædiabetes eller type 2-diabetes<br>○ Sundhedsprofessionel, som arbejder med mennesker med prædiabetes eller type 2-diabetes<br>○ Forsker, som arbejder med prædiabetes eller type 2-diabetes<br>○ Andet? (Uddyb venligst i nedenstående)
Uddyb venligst	____
Hvilken type sundhedsprofessionel er du?	○ Praktiserende læge<br>○ Sygeplejerske<br>○ Diætist<br>○ Andet
Uddyb venligst hvilken type sundhedsprofessionel du er	____
Uddyb venligst hvilken forskning du udfører	____
Hvornår fik du diagnosticeret type 2-diabetes?	○ for 0-1 år siden<br>○ for 1-2 år siden<br>○ for 2-5 år siden<br>○ for 5-10 år siden<br>○ for 10-20 år siden<br>○ for + 20 år siden

Hvor bor du?	◯ Region Nordjylland<br>◯ Region Midtjylland<br>◯ Region Syddanmark<br>◯ Region Sjælland<br>◯ Region Hovedstaden
Hvor arbejder du?	◯ Region Nordjylland<br>◯ Region Midtjylland<br>◯ Region Syddanmark<br>◯ Region Sjælland<br>◯ Region Hovedstaden
Hvad er din alder?	◯ 18-30 år<br>◯ 31-50 år<br>◯ 51-70 år<br>◯ 71+ år
Hvad er dit køn?	◯ Kvinde<br>◯ Mand<br>◯ Foretrækker ikke at sige<br>◯ Foretrækker at beskrive selv

Uddyb venligst

____

Hvilke(t) spørgsmål om prædiabetes vil du gerne have besvaret af forskning?

Tænk gerne bredt over enhver behandling, strategi, organisering af sundhedsvæsenet eller andet som du vurderer, er vigtigt at forske i. Dine spørgsmål kunne f.eks. omhandle diagnose, risikofaktorer, livsstilsinterventioner, behandlingsmuligheder, langsigtede sundhedskonsekvenser, forbyggelse af type 2-diabetes eller noget helt andet.

Prøv gerne at beskriv præcise spørgsmål, snarere end generelle kommentarer.

Du må gerne beskrive 5 forskellige spørgsmål, men mindst 1 spørgsmål er påkrævet for at gennemføre spørgeskemaet.

Hvad vil du gerne have besvaret af forskning?

Beskriv din 1. prioritet her: ____________________

Hvad vil du gerne have besvaret af forskning?

Beskriv din 2. prioritet her: ____________________

Hvad vil du gerne have besvaret af forskning?

Beskriv din 3. prioritet her: ____________________

Hvad vil du gerne have besvaret af forskning?

Beskriv din 4. prioritet her: ____________________

Hvad vil du gerne have besvaret af forskning?

Beskriv din 5. prioritet her: ____________________

**Afslutning**	
Har du lyst til deltage i fremtidige faser af dette projekt eller fremtidige projekter relateret til prædiabetes?	☐ Nej tak<br>☐ Ja tak - jeg vil gerne deltage i fremtidige faser<br>☐ Ja tak - jeg vil gerne deltage i fremtidige projekter<br>((Flere svar kan vælges))
Skriv venligst din email og telefon nummer, hvis vi må kontakte dig angående videre involvering.<br><br>E-mail	____________________<br>(E-mail)
Telefon nummer	____________________<br>(Telefon nummer)

ESM Figure 1: Survey questionnaire used to gather uncertainties in the first step of the priority setting partnership.

ESM Figure 2

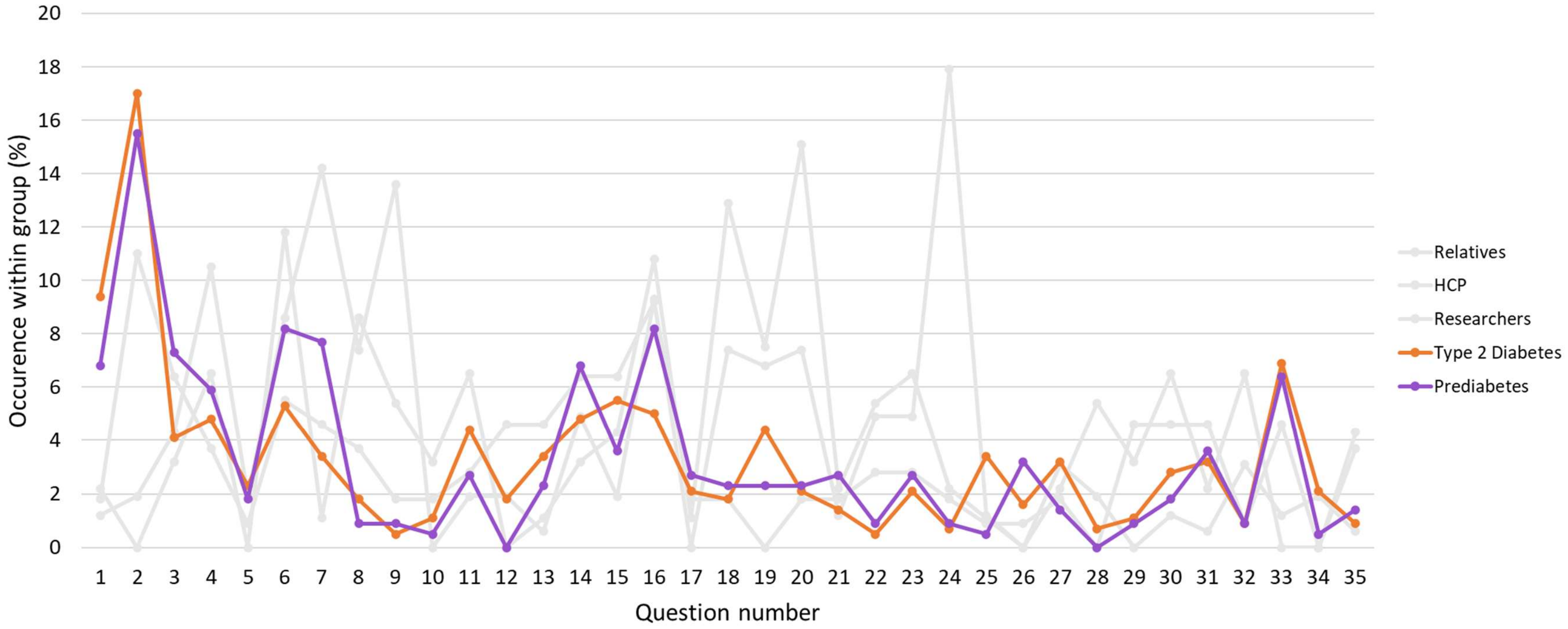

ESM Figure 2: Sensitivity analysis between prediabetes and type 2 diabetes participants from survey 1 (Step 1 in the PSP process). Occurrence within group represents how many percent of the participants submitted an uncertainty within the specific sub-theme.

ESM Figure 3

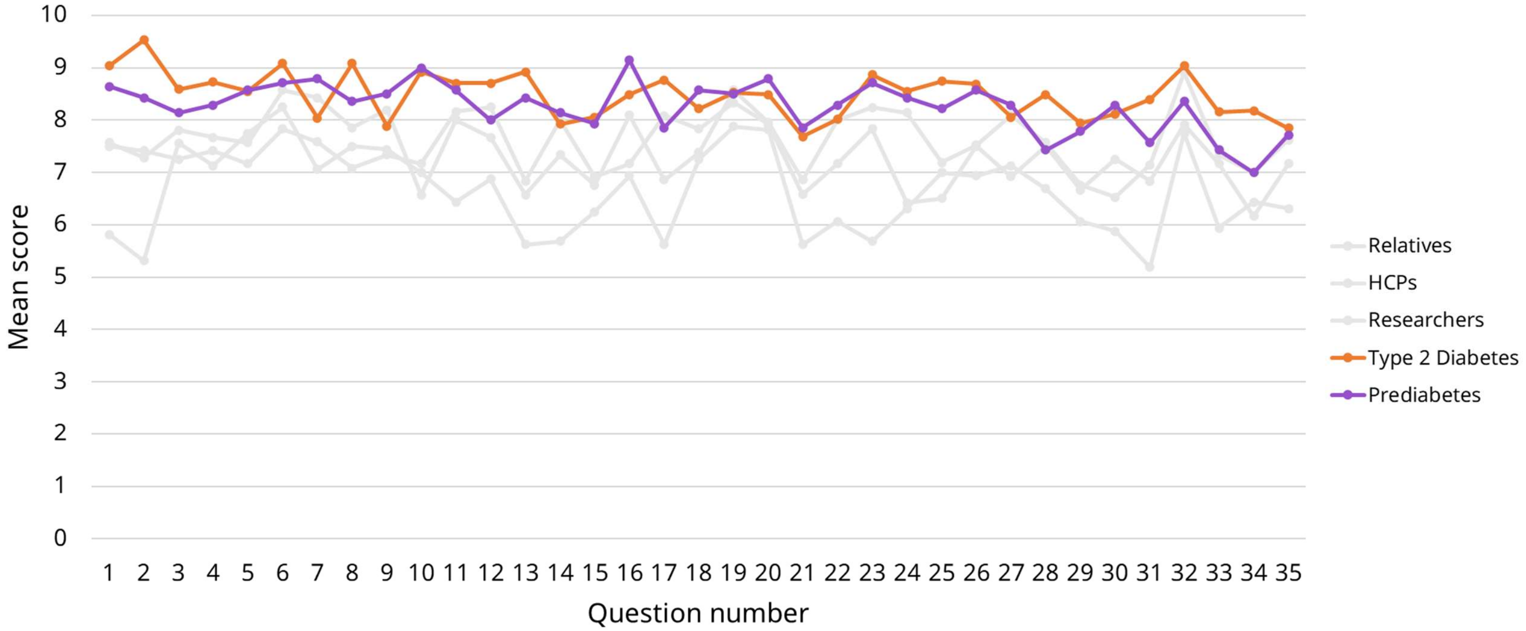

ESM Figure 3: Sensitivity analysis between prediabetes and type 2 diabetes participants from survey 2 (Step 3 in the PSP process). Participants scored each indicative question from 1 to 10; with 1 = Not important at all and 10 = very important.

ESM Figure 4

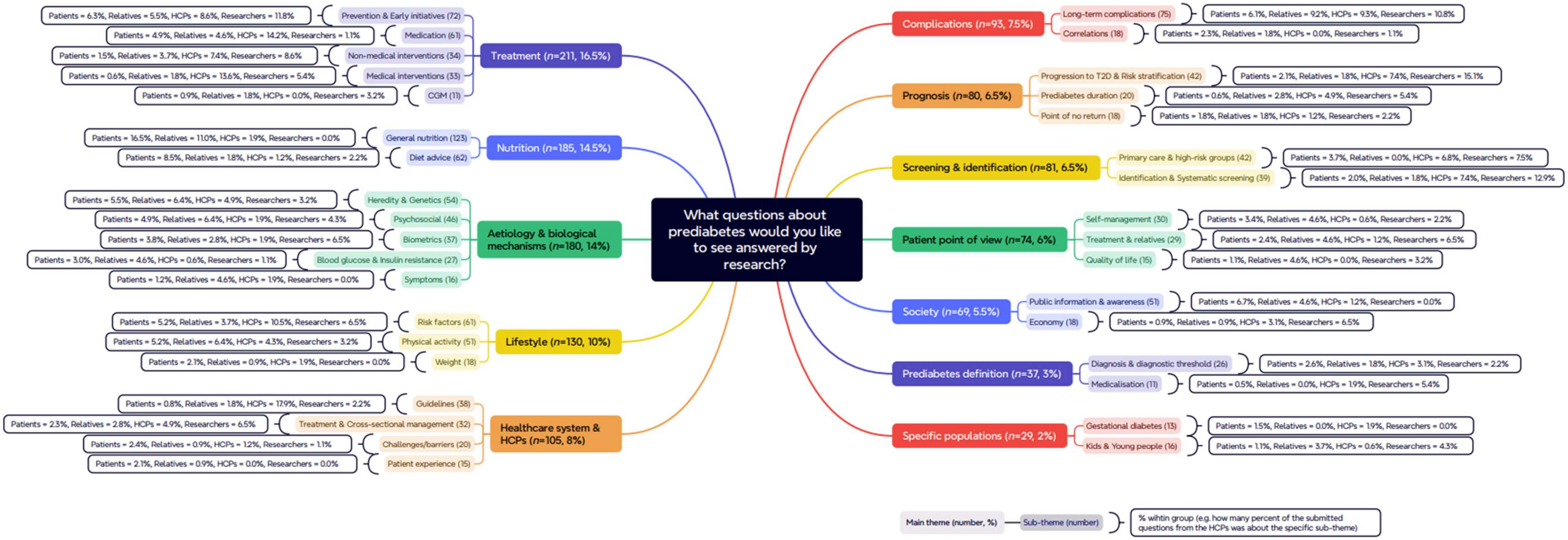

ESM Figure 4: The 12 main themes and 35 sub-themes with information about how many times each main theme and sub-theme was mentioned in the collected uncertainties. Furthermore, it includes information on how many percent of the participants within each group mentioned the specific sub-theme.
